# Differences Between Pediatric and Adult T Cell Responses to *In Vitro* Staphylococcal Enterotoxin B Stimulation

**DOI:** 10.3389/fimmu.2018.00498

**Published:** 2018-03-20

**Authors:** Mark E. Rudolph, Monica A. McArthur, Robin S. Barnes, Laurence S. Magder, Wilbur H. Chen, Marcelo B. Sztein

**Affiliations:** ^1^Center for Vaccine Development, University of Maryland School of Medicine, Baltimore, MD, United States; ^2^Molecular Microbiology and Immunology Department, University of Maryland Graduate Program in Life Sciences, Baltimore, MD, United States; ^3^Department of Pediatrics, University of Maryland School of Medicine, Baltimore, MD, United States; ^4^Epidemiology and Public Health, University of Maryland School of Medicine, Baltimore, MD, United States; ^5^Department of Medicine, University of Maryland School of Medicine, Baltimore, MD, United States

**Keywords:** pediatric immunology, T cell response, staphylococcal enterotoxin B, toxic shock syndrome, multifunctionality, mass cytometry, dimensionality reduction

## Abstract

Toxic shock syndrome (TSS) is capable of inducing life-threatening fever, rash, and systemic organ failure, though the specific mechanisms behind these symptoms remain poorly understood. Staphylococcal enterotoxin B (SEB) and other superantigens have shown to be important factors in TSS, capable of promoting cross-linking between T cell receptors and major histocompatibility complexes which results in overwhelming T cell activation, proliferation, and cytokine production. The resulting proinflammatory cytokine cascade, often referred to as the “cytokine storm,” seems to be critical to the development of disease. Interestingly, clinical studies have shown that children exhibit less severe TSS-associated morbidity than adults, though the mechanism behind this phenomenon has not been addressed. Indeed, despite the fact that most novel antigen exposure occurs early in life, be it from environmentally acquired pathogens or routine vaccination, normal pediatric T cell immune functions remain critically underexplored. This is largely due to difficulty in obtaining enough samples to explore more than a narrow sliver of the cell-mediated immune compartment. To address this limitation, we optimized a T effector (T_eff_)/circulating T follicular helper (cT_FH_) cell mass cytometry panel which allowed us to analyze a wide array of T cell populations and effector functions following *in vitro* SEB stimulation. We show that T cell activation—as measured by CD69 expression—following SEB stimulation is lower in pediatric participants, increasing throughout childhood, and reaching adult levels by around 15 years old. Further, while individual CD4^+^ effector memory T cell (T_EM_) effector molecules show limited age-associated differences following SEB stimulation, multifunctional CD4^+^ T_EM_ are shown to positively correlate with increasing age through adolescence. Individual CD8^+^ T_EM_ effectors and multifunctional phenotypes also show very strong age-associated increases following SEB stimulation. SEB stimulation has little impact on cT_FH_ activation or functional cellular markers, regardless of age. These results, coupled with the fact that a robust proinflammatory cytokine response seems critical to developing severe TSS, suggest a possible connection between the significantly reduced T cell activation and multifunctional populations following *in vitro* SEB stimulation in our pediatric participants and clinical observations relating to reduced TSS mortality in children.

## Introduction

Superantigens are molecules produced by viral and bacterial pathogens which are capable of inducing massive non-specific, polyclonal T cell activation and effector molecule release. They accomplish this by bypassing canonical antigen processing and directly linking multiple major histocompatibility complex (MHC) class II molecules to the T cell receptor (TCR)-β subunit outside of the traditional antigen binding groove (1). Further, superantigens bind the costimulatory CD28 homodimer at the immunological synapse, thus providing a second signal, necessary to drive robust T cell proliferation, activation, and effector molecule production (2). The resulting non-clonal specific inflammatory T cell responses can activate more than 20% of the peripheral T cell population (1–4). Further, the induced proinflammatory response leads to the recruitment and expansion of a variety of lymphocytes, including innate immune cells such as macrophages and neutrophils. These innate immune cells contribute to the proinflammatory milieu while also providing the system with additional MHC through which superantigen can bind to and activate more TCRs (3). Staphylococcal enterotoxin B (SEB) is known to be associated with food poisoning (preformed staphylococcal enterotoxin ingestion) and non-menstrual toxic shock syndrome (TSS), including soft-tissue associated infections (4). TSS is a severe, sometimes fatal disease that results from the so called “cytokine storm” which follows superantigen TCR/MHC cross-linking; however, the mechanism by which SEB induces TSS is not fully understood. Proinflammatory cytokines are known to be important to TSS pathogenesis, and SEB stimulation of T cells can induce the expression of multiple CD4^+^ and CD8^+^ T_EM_-associated effector molecules, including tumor necrosis factor (TNF)-α, interleukin (IL)-2, IL-17A, interferon (IFN)-γ, macrophage inflammatory protein (MIP)-1β, the degranulation marker CD107a, and Granzyme B (3–6). Interestingly, TSS-associated mortality has been shown to be less severe in children than in adults (7), but the mechanism of this observed attenuated phenotype has remained unexplored.

Healthy pediatric immune responses are difficult to study, due mostly to limited sample accessibility. However, because many pathogens, as well as vaccinations, are encountered early in life, it is critically important to better understand pediatric and adolescent T cell responses. Limited data have shown notable T cell population differences and similarities between adults and children. In general, the percentage of total T cells (CD3^+^ lymphocytes) and CD8^+^ T cells among peripheral blood mononuclear cells (PBMC) remain fairly constant from childhood into adulthood, while the percentage of CD4^+^ T cell increases throughout life (8, 9). Observations of memory T cell populations show higher naive T cell proportions in children, with memory T cell phenotypes increasing into and through adulthood (9–11). Further, TCR-β repertoire diversity decreases as individuals age (12). Most research on pediatric T cell effector function is limited to observing responses to vaccination against common viral pathogens. Data that have been collected show a few age-related differences among individual effector cytokine responses, but no over-arching age-associated patterns have emerged (13–15). It has been shown, however, that vaccination of younger children is less likely to induce lasting protective cell-mediated immune (CMI) functions, including T follicular helper (TFH) responses, than in older children and adults (16, 17). Interestingly, in a study that included twelve healthy pediatric participants between the ages of 7 and 17 years old, mitogen-activated CD8^+^ T_EM_ cell multifunctionality was observed to be less robust in children than in adults (18).

Multifunctional T cells are defined as individual T cells which simultaneously exhibit multiple effector functions, such as degranulation, as well as the production of cytokines/chemokines. Multifunctionality has been observed in memory CD4^+^ and CD8^+^ T cells following stimulation with their cognate antigen, as well as after superantigen stimulation. Effector expression can vary within multifunctional T cells, but previous work from our group has shown a robust IL-2, IFN-γ, and TNF-α triple-positive phenotype within CD8 effector memory T cells following SEB stimulation in adult PBMC (6). Multifunctional CD4^+^ and CD8^+^ T cells are important in controlling a variety of viral and bacterial pathogens (19–22). Additionally, there are data that suggest that multifunctional T cells are more frequently observed in the elderly following SEB stimulation, compared with younger adults (23). However, beyond preliminary data from our group (18), as far as we know, there are no reports that have evaluated in great detail multifunctional T cell immune responses in children.

To better explore differences between pediatric and adult multifunctional T cell responsiveness, we utilized an *in vitro* SEB stimulation protocol and analyzed the responses by multiparameter mass cytometry. Our mass cytometry panel was developed to enable us to observe T cell populations, memory, activation, and proliferation status, as well as production of multiple cytokines/chemokines. Here, we describe how age-associated differences in T cell multifunctionality and disparities among how various T cell subsets respond to SEB stimulation may play a role in the previously reported disparity of TSS clinical outcome between children and adults, and more generally how these differences may prove critical to any future pediatric vaccination and treatment strategies.

## Materials and Methods

### Participants and Isolation of PBMC

Peripheral blood mononuclear cells were collected from 20 healthy pediatric (6–17 years of age at the time of enrollment) and 14 healthy adult (20–65 years of age at the time of enrollment) volunteers, being recruited from the Baltimore-Washington area and the University of Maryland at Baltimore campus. These studies were approved by the University of Maryland at Baltimore Institutional Review Board and were carried out in accordance with the Declaration of Helsinki. Written informed consent was obtained from all adult participants, as well as written assent and informed consent from the parents of any participant under the age of 18 years old—and assent from the pediatric participants themselves—prior to the conduct of any study procedures. PBMC were isolated immediately following blood collection, by density gradient centrifugation, and were then stored in liquid nitrogen following standard cryopreservation techniques (24, 25), until ready for use.

### *In Vitro* Stimulation

Peripheral blood mononuclear cell were thawed and rested overnight at 37°C, 5% CO_2_ in RPMI 1640 media (Gibco, Carlsbad, CA, USA), supplemented with 100 U/mL penicillin (Sigma), 100 µg/mL streptomycin (Sigma, St. Louis, MO, USA), 50 µg/mL gentamicin (Gibco), 2 mM l-glutamine (Gibco), 2.5 mM sodium pyruvate (Gibco), 10 mM HEPES buffer (Gibco), non-essential amino acids (Lonza, Basel, Switzerland), and 10% fetal bovine serum (Gemini Bioproducts, West Sacramento, CA, USA) to make complete RPMI (cRPMI). After overnight rest, cells were washed and resuspended in cRPMI at a concentration of 1 × 10^6^ cells/500 μL in 5 mL in cell culture tubes and incubated with SEB (Toxin Technology, Sarasota, FL, USA) at 10 µg/mL and 3 µl/mL anti-CD107a monoclonal antibody (mAb) conjugated to 151Eu (Fluidigm, South San Francisco, CA, USA) for 2 h at 37°C in 5% CO_2_. Media (cRPMI with 3 µl/mL anti-CD107a-151Eu mAb) was used as a negative control. After the 2-h incubation, GolgiStop (containing monensin) and GolgiPlug (containing brefeldin A) from BD (San Jose, CA, USA) were added at 0.5 µl/mL to all tubes and cultures were maintained at 37°C in 5% CO_2_ overnight.

### Surface and Intracellular Labeling and Mass Cytometry Analysis

Following stimulation, PBMC were spun down and incubated with anti-CD45 (Fluidigm South San Francisco, CA, USA) mAb for barcoding. Pediatric samples were stained with CD45-154Sm and adult samples were stained with CD45-156Gd for 30 min at 4°C. Cells were then washed once with flow buffer [1× PBS (Quality Biological, Gaithersburg, MD, USA), 0.1% sodium azide (Sigma), and 2% fetal bovine serum (Gemini Bioproducts)] and once with serum-free RPMI (Gibco) before being combined into their barcoded layout. Like-stimulated adult (CD45-156Gd) and pediatric (CD45-154Sm) PBMC were combined into a single tube for downstream staining. Mass cytometry staining was performed as described in the Materials and Methods section “Mass Cytometry Measurements” from McArthur (26) using the monoclonal antibodies listed in Table 1. In brief, cells were labeled with metal-tagged antibodies against specific surface and intracellular targets, as well as with cisplatin as a viability marker and iridium as a DNA intercalator, before being prepared and run by our flow and mass cytometry core in a CyTOF instrument (Fluidigm). Mass cytometry data were analyzed using WinList version 9.0.1 (Verity Software House, Topsham, ME, USA) following debarcoding of the files with Premium Cytobank (Cytobank, Inc., Santa Clara, CA, USA) (Figure S1 in Supplementary Material). CITRUS analyses were performed in Premium Cytobank.

**Table 1:** Mass cytometry panel showing antibody target, stable metal isotope (or other label), antibody clone, and a brief description of the target function.

Target	Stable metal isotope	Clone	Description
CD14	114 Cd (Qdot)	TüK4	Monocyte marker
CD19	114 Cd (Qdot)	SJ25-C1	B cell marker
*CXCR5*	*Biotin*	*RF8B2*	Follicular homing chemokine receptor
Biotin	143 Nd	1D4-C5	
CD8	146 Nd	RPA-T8	Cytotoxic T lymphocyte marker
MIP-1β	150 Nd	D21-1351	NK and monocyte recruiting chemokine
CD107a	151 Eu	H4A3	Degranulation marker
TNF-α	152 Sm	Mab11	Proinflammatory cytokine
CD62L	153 Eu	DREG-56	Lymphoid-tissue homing selectin
CD45	154 Sm	HI30	Pan-leukocyte barcoding marker
CD27	155 Gd	L128	TNF superfamily—costimulatory molecule
CD45	156 Gd	HI30	Pan-leukocyte barcoding marker
IL-2	158 Gd	MQ1-17H12	Induction of T cell differentiation and proliferation
Ki67	161 Dy	B56	Nuclear proliferation marker
CD69	162 Dy	FN50	Activation marker
IL-17A	164 Dy	N49-653	Tc/h17 effector cytokine
IFN-γ	165 Ho	B27	Proinflammatory cytokine
IL-10	166 Er	JES3-9D7	Anti-inflammatory cytokine
CD154 (CD40L)	168 Er	24-31	Costimulatory molecule; Tfh induction of B cell maturation
CD45RA	169 Tm	HI-100	T cell memory marker
CD3	170 Er	UCHT1	TCR coreceptor (T cell marker)
Granzyme B	171 Yb	GB11	Secreted cytotoxic effector molecule
IL-21	172 Yb	3A3-N2	Tfh effector cytokine (germinal center formation)
ICOS (CD278)	173 Yb	C398.4A	Tfh costimulatory marker (B cell help)
CD4	174 Yb	SK3	Helper T lymphocyte marker
Cell I.D. (DNA)	191/193 Ir	n/a	DNA intercalator
Viability	194/195 Pt	n/a	Viability stain

*Primary surface antibodies are filled with blue, secondary surface antibodies with green, and intracellular antibodies with orange. Barcoding antibodies are shaded in yellow, and anti-CD107a, which is added during stimulation to best account for the active cycling between the surface and intracellular vesicles, is highlighted in green*.

### Statistical Analyses

All analyses, except for CITRUS, were performed using GraphPad Prism version 7.0. Unpaired *t*-tests or Spearman’s correlations were performed depending on the analysis as indicated. *p*-Values of <0.05 were considered significant.

## Results

### Age-Dependent Variability Among Baseline T Cell Populations

**Figure 1:**
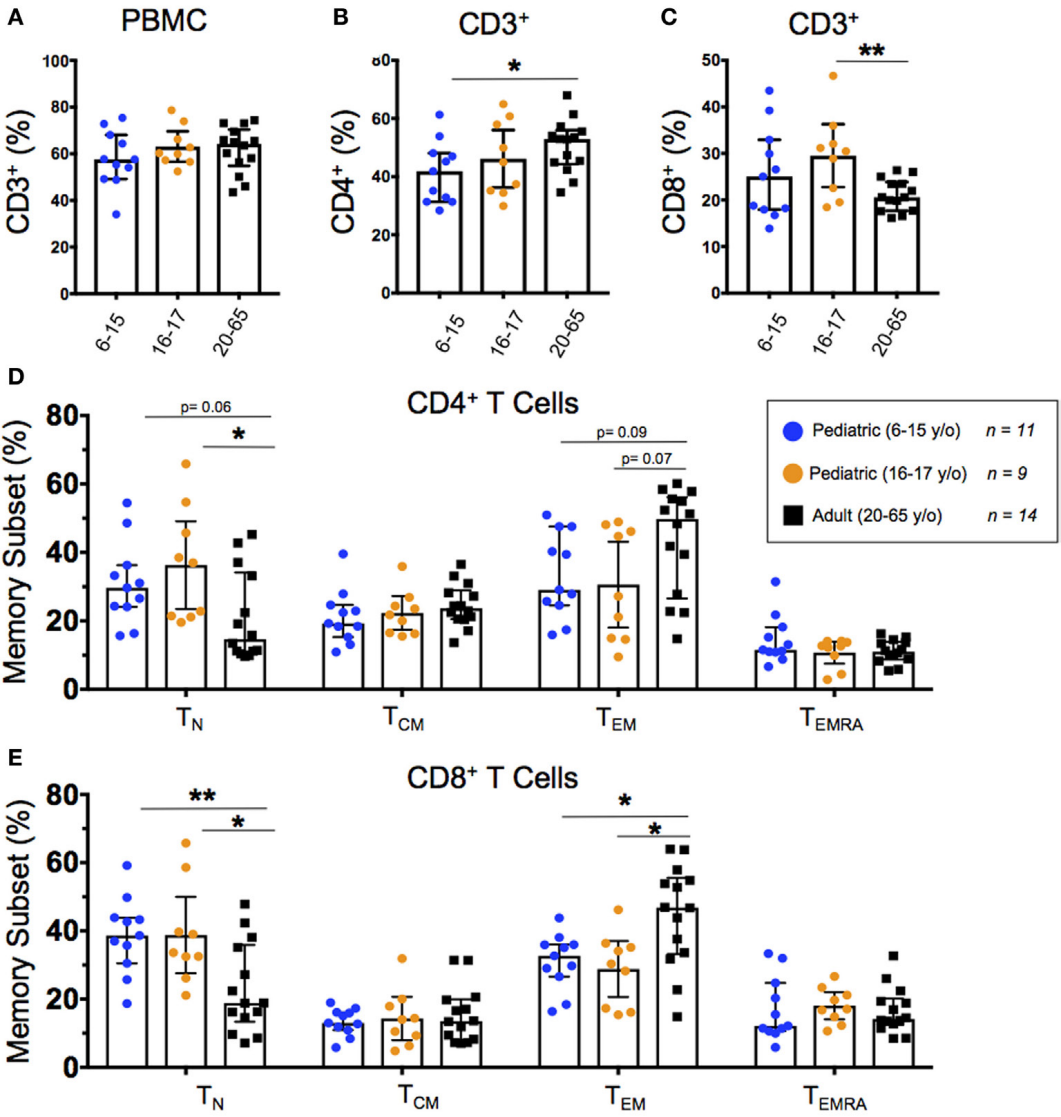
Baseline T cell populations. Scatter plots showing the percentages of **(A)** baseline CD3^+^ T cells, **(B)** CD3^+^CD4^+^ helper T cells, and **(C)** CD3^+^CD8^+^ cytotoxic T cells, as well as **(D)** CD4^+^ and **(E)** CD8^+^ naive (CD45RA^+^CD62L^+^), T central memory (T_CM_; CD45RA^−^CD62L^+^), effector memory (CD45RA^−^CD62L^−^), and EMRA (CD45RA^+^CD62L^−^) populations among 6–15-year-old pediatric (*n* = 11), 16–17-year-old pediatric (*n* = 9), and adult (*n* = 14) participants (media). Bars represent medians with whiskers indicating interquartile ranges. Statistics were analyzed by unpaired *t*-test (*p < 0.05; **p < 0.01).

We stained unstimulated PBMC from healthy pediatric and adult participants with metal-conjugated monoclonal antibodies followed by acquisition on a mass cytometer (Table 1). Total T cells (CD3^+^CD14^−^CD19^−^) were subdivided into helper T cells (T_H_; CD4^+^CD8^−^) and cytotoxic T cells (T_C_; CD4^−^CD8^+^) populations. We further used expression of CD62L and CD45RA to define T cell memory subsets including naive (T_N_; CD62L^+^CD45RA^+^), central memory (T_CM_; CD62L^+^CD45RA^−^), effector memory (T_EM_; CD62L^−^CD45RA^−^), and effector memory CD45RA^+^ (T_EMRA_; CD62L^−^CD45RA^+^). The identification of T memory subsets using the CD62L/CD45RA classification is an established methodology, as CD62L^−/lo^ populations map well with effector memory as defined by CCR7 (27). In order to better identify differentiated memory T cells, CD27 is included in our panel (28–30). T cell populations and memory subsets were compared among 6–15-year-old pediatric (*n* = 11), 16–17-year-old pediatric (*n* = 9), and adult (20–65 year-old; *n* = 14) participants (Figure 1).

All 34 participants showed similar total T cell percentages regardless of age (Figure 1A). As previously described (8, 9), we observed a relationship between age and T_H_ with a significantly lower percentage of CD4^+^ T cells in younger pediatric participants compared to adults (Figure 1B). Although 16–17-year-old pediatric participants had a significantly higher percentage of CD8^+^ T cells than adults, the trend did not extend to the younger pediatric participants (Figure 1C). The CD8^+^ T_N_ and T_EM_ subsets differed significantly according to age (and trended among CD4^+^) with younger participants demonstrating a higher percentage of T_N_ and a lower percentage of T_EM_ (Figures 1D,E). However, T_CM_ and T_EMRA_ did not show any significant age-dependent trends in either CD4^+^ or CD8^+^ populations (Figures 1D,E). Furthermore, when we graphed percent memory subsets as a function of age, T_N_ cells consistently decreased with age while T_EM_ consistently increased with age (Figures S2A,B in Supplementary Material).

### T Cell Activation Following SEB Stimulation Increases Throughout Childhood

**Figure 2:**
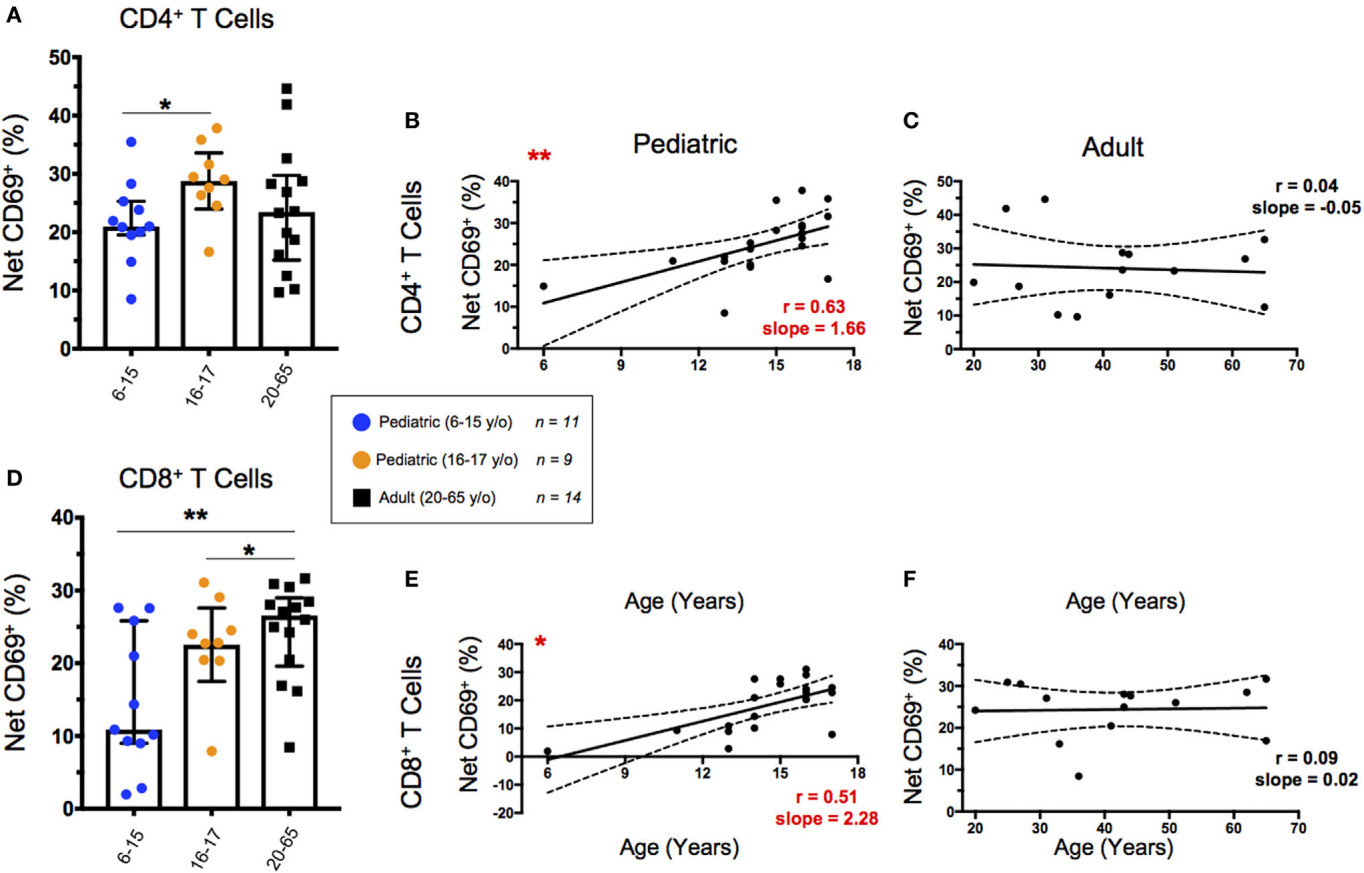
CD69 expression following staphylococcal enterotoxin B (SEB) stimulation. Scatter plots showing the percentages of CD69^+^ in **(A)** CD4^+^ and **(D)** CD8^+^ T cells. Net CD69^+^ cells represent the % CD69^+^ cells in SEB-stimulated cultures minus expression in the corresponding media controls. Populations are shown for 6–15-year-old pediatric (*n* = 11), 16–17-year-old pediatric (*n* = 9), and adult (*n* = 14) participants. Bars represent medians with whiskers indicating interquartile ranges. Scatter plot statistics were analyzed by unpaired *t*-test (*p < 0.05; **p < 0.01). Line graphs represent linear regression with 95% confidence intervals among net CD69^+^ CD4^+^ **(B,C)** and CD8^+^ **(E,F)** T cell populations in participants between the ages of 6–17 [**(B,E)**; *n* = 20] and 20–65 [**(C,F)**; *n* = 14] years old. Line graph statistics were analyzed by Spearman *r* correlation (*p < 0.05; **p < 0.01).

We used CD69 expression to define activated T cell populations following SEB stimulation. To calculate net expression, we subtracted the percentages of CD69^+^ cells in media controls from the percentages of CD69^+^ cells in SEB-stimulated cultures. Net activation of CD4^+^ T cells was significantly lower in younger pediatric participants than in older pediatric participants; however, there were no significant differences between the pediatric age groups and the adult participants (Figure 2A). When we focused on net activation following SEB stimulation as a function of age within the pediatric participants, there was a significant increase of net CD69 expression to approximately 25% of CD4^+^ T cells (Figure 2B). In contrast, SEB-induced CD69 expression remained consistent throughout adulthood (Figure 2C). CD8^+^ T cells demonstrated significant increases in net CD69 expression among both pediatric age strata compared to adults (Figure 2D) and as a function of aging throughout childhood (Figure 2E). As with activated CD4^+^ T cells, net activation of CD8^+^ T cells by SEB stimulation reached adult levels (~25%) by around the age of 15 years and are maintained throughout adulthood (Figure 2F).

### SEB Stimulated CD4^+^ T_EM_ Show Increased Functionality Throughout Childhood

**Figure 3:**
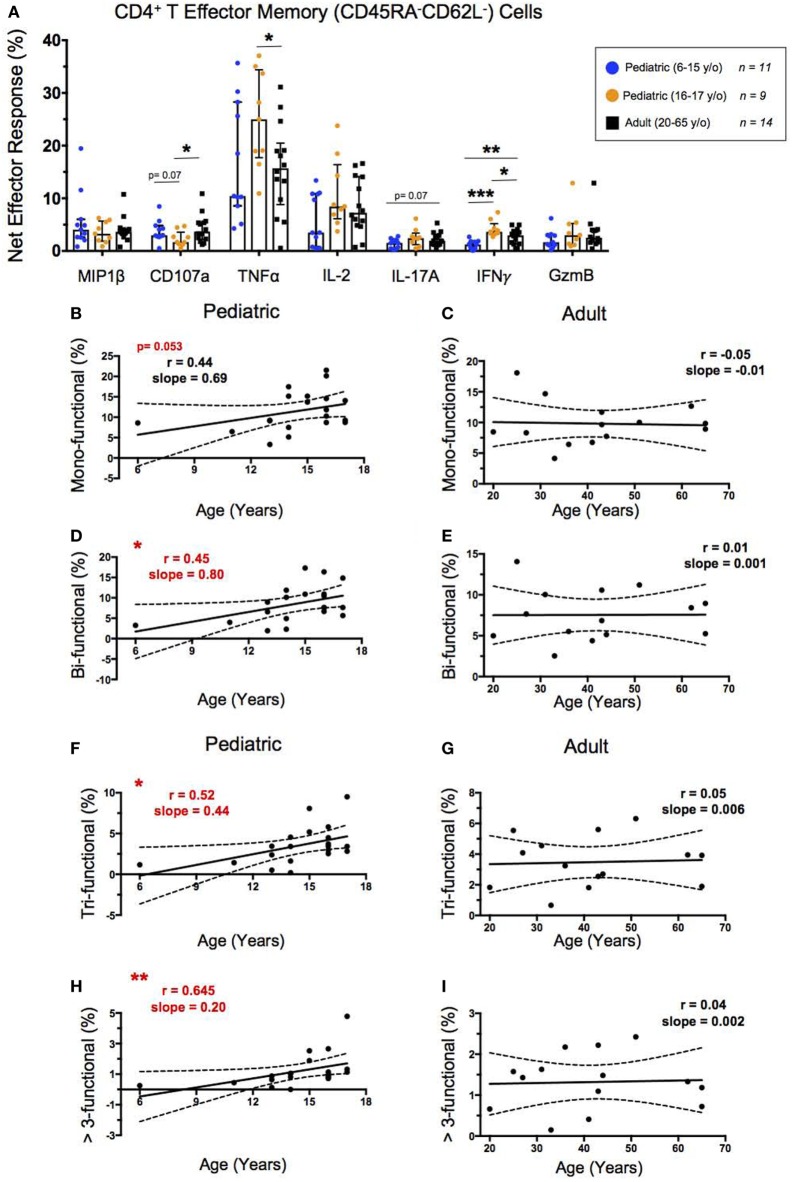
CD4^+^ T effector memory response. **(A)** Scatter plot showing the net percentages of CD4^+^ T_EM_ producing MIP-1β, CD107a, TNF-α, IL-2, IL-17A, IFN-γ, and ***Granzyme B (GzmB) following SEB stimulation. Results are shown for the following populations: 6–15-year-old pediatric (*n* = 11), 16–17-year-old pediatric (*n* = 9), and adult (*n* = 14) participants. Bars represent medians with whiskers indicating interquartile ranges. Scatter plot statistics were analyzed by unpaired *t*-test (*p < 0.05; **p < 0.01; ***p < 0.001). Line graphs represent linear regression with 95% confidence intervals among CD4^+^ T effector memory cell mono- **(B,C)**, bi- (**D,E)**, tri- **(F,G)**, and >3- **(H,I)** functional populations following SEB stimulation within participants between the ages of 6–17 (*n* = 20) and 20–65 (*n* = 14) years old. Line graph statistics were analyzed by Spearman *r* correlation (*p < 0.05; **p < 0.01).

To explore the functionality of CD4^+^ T_EM_, we first determined the percent net expression of effector molecules MIP-1β, CD107a, TNF-α, IL-2, IL-17A, IFN-γ, and Granzyme B following SEB stimulation (Figure 3A). Of these effector functions, IFN-γ demonstrated a strongly significant age-dependence with lower levels of net IFN-γ expression in younger pediatric participants than both the older pediatric and adult participants. IL-17A also trended toward an age-dependent increase in net expression following SEB stimulation but did not reach statistical significance (*p* = 0.07). Interestingly, older pediatric participants showed significantly greater TNF-α and IFN-γ expression than adults, as well as lower CD107a expression than adults and younger children.

We used the FCOM™ feature of WinList version 9.0.1 to explore all possible combinations of the seven aforementioned biomarkers. These results were used to tabulate the percentages of CD4^+^ T_EM_ that expressed 1, 2, 3, or >3 of the measured effector functions to determine whether increased functionality is impacted by age. Similar to our previous age-dependent observations of CD4^+^ T_EM_ activation by net CD69 expression, the net functionality of CD4^+^ T_EM_, as characterized by MIP-1β, CD107a, TNF-α, IL-2, IL-17A, IFN-γ, and Granzyme B, was lower in younger children, but increased to adult levels by ~15 years (Figures 3B–I). The CD4^+^ T_EM_ expressing a single measured effector function showed an increase from the age of 6–17 that approached statistical significance (*p* = 0.053) (Figure 3B), whereas CD4^+^ T_EM_ expressing 2, 3, or >3 effector functions all showed direct, significant correlations between increasing age through adolescence and multifunctional responses (Figures 3D,F,H). In contrast, in adults, net functional responses did not correlate with increasing age, maintaining similar levels from 20 to 65 years of age (Figures 3C,E,G,I).

### SEB Stimulated CD8^+^ T_EM_ Effector Responses and Functionality Increase Significantly Throughout Childhood

**Figure 4:**
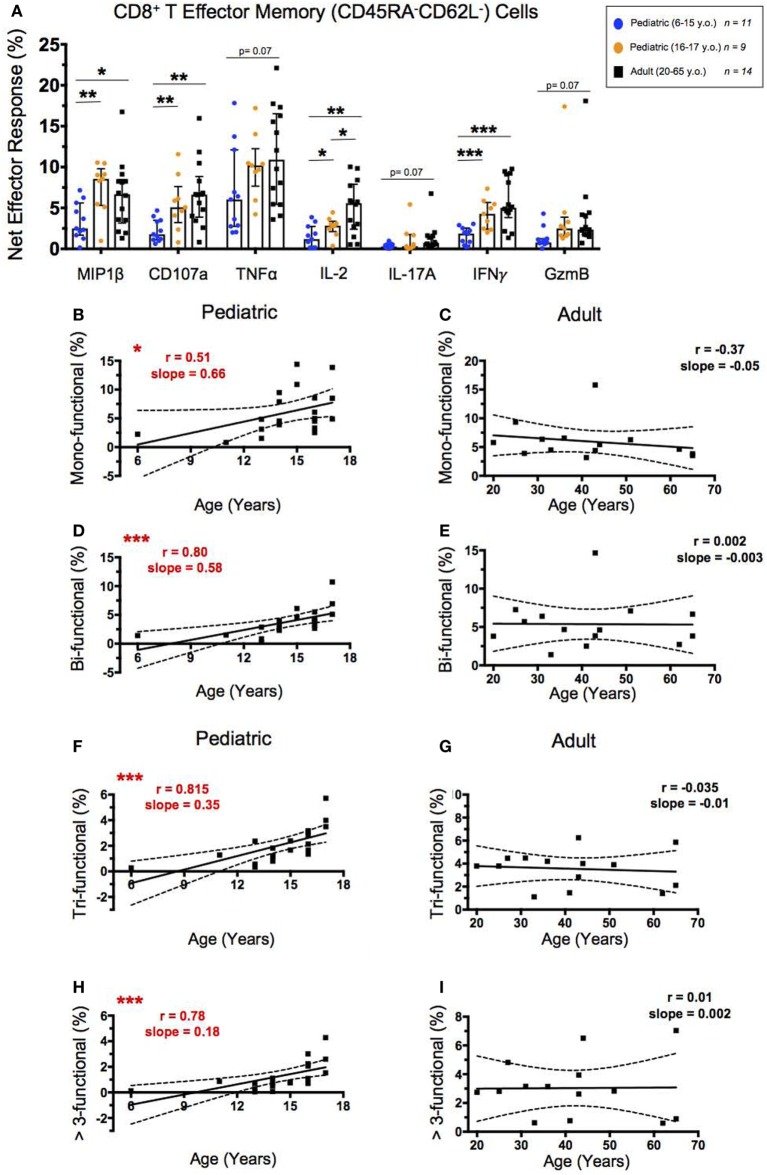
CD8^+^ T effector memory response. **(A)** Scatter plot showing the net percentages of CD8^+^ T_EM_ producing MIP-1β, CD107a, TNF-α, IL-2, IL-17A, IFN-γ, and Granzyme B (GzmB) following SEB stimulation. Results are shown for the following populations: 6–15-year-old pediatric (*n* = 11), 16–17-year-old pediatric (*n* = 9), and adult (*n* = 14) participants. Bars represent medians with whiskers indicating interquartile ranges. Scatter plot statistics were analyzed by unpaired *t*-test (*p < 0.05; **p < 0.01; ***p < 0.001). Line graphs represent linear regression with 95% confidence intervals among CD8^+^ T effector memory cell mono- **(B,C)**, bi- **(D,E)**, tri- **(F,G)**, and >3- (**H,I)** functional populations following SEB stimulation within participants between the ages of 6–17 (*n* = 20) and 20–65 (*n* = 14) years old. Line graph statistics were analyzed by Spearman *r* correlation (*p < 0.05; ***p < 0.001).

To analyze the functionality of CD8^+^ T_EM_, we again looked at the percent net expression of effector molecules MIP-1β, CD107a, TNF-α, IL-2, IL-17A, IFN-γ, and Granzyme B following SEB stimulation (Figure 4A). In contrast to the majority of the CD4^+^ T_EM_ effector responses, CD8^+^ T_EM_ net MIP-1β, CD107a, and IL-2 expression, in addition to IFN-γ expression, were significantly higher in adults than in younger participants following SEB stimulation. Further, while the net effector responses between the older pediatric (16–17 years old) and adult participants differed less, significant age dependence was seen with IL-2 expression.

We again used FCOM™ to explore percentages of CD8^+^ T_EM_ that expressed 1, 2, 3, or >3 of the measured effector functions to determine whether increased functionality correlated with age. CD8^+^ T_EM_ expressing 1, 2, 3, or >3 of the measured effector functions all showed direct significant correlations between increasing age through adolescence and percent net functionality, with the most significant correlations demonstrated by the populations expressing >1 effector function (Figures 4B,D,F,H). Similar to CD4^+^ T_EM_, the adult net functional CD8^+^ T_EM_ responses showed no correlation between age and functionality and maintained similar levels from 20 to 65 years of age (Figures 4C,E,G,I).

### Gender Is Not Associated With Major Differences in CD4^+^ and CD8^+^ T_EM_ Effector Responses and Functionality Following SEB Stimulation

To explore whether gender may play a role in the previously observed heterogeneous responses to SEB, we compared CD4^+^ and CD8^+^ T_EM_ effector responses between male and female participants. As our two youngest pediatric participants were both male (ages 6 and 11, respectively) and likely prepubescent, we excluded them from these analyses. We further performed gender comparisons between like-age groups to avoid confounding gender- and age-associated heterogeneity. Pediatric males had higher net CD4^+^ T_EM_ expression of TNF-α, IL-2, and IFN-γ than pediatric females (Figure S3A in Supplementary Material). However, there were no significant gender-associated differences in CD4^+^ T_EM_ expressing 1, 2, 3, or >3 effector functions in the pediatric participants (Figure S3B in Supplementary Material). Of interest, there were no significant gender-associated differences in the CD4^+^ T_EM_ effector functionality among the adult participants (Figures S3C,D in Supplementary Material). While pediatric males demonstrated significantly higher net CD8^+^ T_EM_ IFN-γ expression than pediatric females, there were no significant gender-associated differences in CD8^+^ T_EM_ expressing 1, 2, 3, or >3 effector functions in the pediatric participants (Figures S3E,F in Supplementary Material). Similar to CD4^+^ T_EM_, there were no significant gender-associated differences in CD8^+^ T_EM_ effector functionality among the adult participants (Figures S3G,H in Supplementary Material).

### Circulating T_FH_ Populations and Functions Show Variability Across Ages

**Figure 5:**
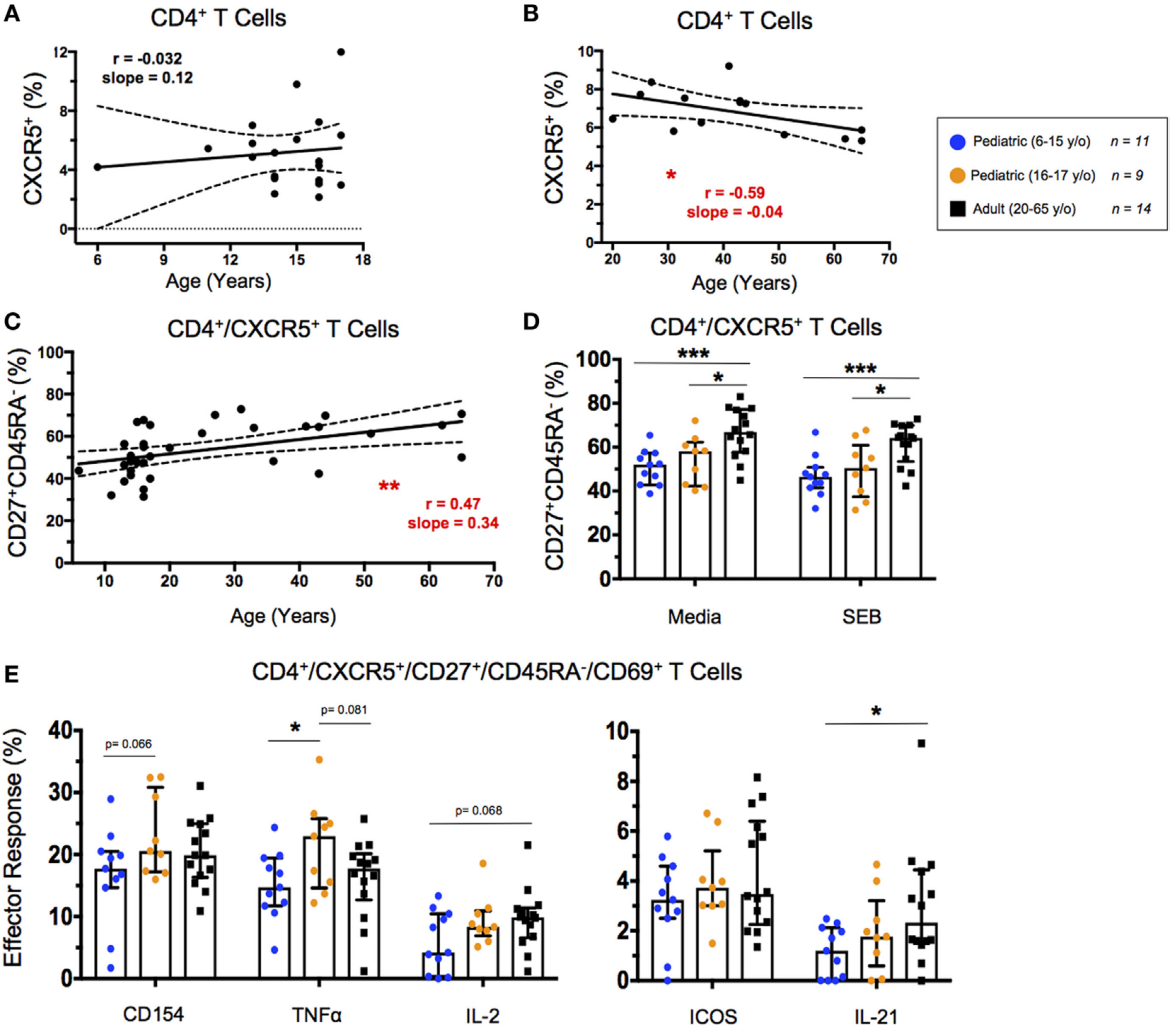
Circulating T follicular helper (cT_FH_) cell response. **(A,B)** Line graphs represent linear regression with 95% confidence intervals among baseline CD4^+^CXCR5^+^ T cells in children between 6 and 17 years old (*n* = 20) and adults between 20 and 65 years old (*n* = 14). **(C)** Line graph represents linear regression with 95% confidence intervals among CD4^+^CXCR5^+^CD27^+^CD45RA^−^ effector cT_FH_ in all volunteers (*n* = 34) between 6 and 65 years old. Line graph statistics were analyzed by Spearman *r* correlation (*p < 0.05; **p < 0.01). **(D)** Scatter plots showing percent effector cT_FH_ under both unstimulated (media) and SEB stimulated conditions. **(E)** Scatter plots showing net percentages of effector cT_FH_ producing CD154 (CD40L), TNF-α, IL-2, ICOS, and IL-21. Populations are shown for 6–15-year-old pediatric (*n* = 11), 16–17-year-old pediatric (*n* = 9) and adult (*n* = 14) participants. Bars represent medians with whiskers indicating interquartile ranges. Scatter plot statistics were analyzed by unpaired *t*-test (*p < 0.05; ***p < 0.001).

The percentages of circulating T_FH_ cells (cT_FH_), defined by CXCR5 expression on CD4^+^ T cells, did not change significantly throughout childhood (Figure 5A). However, cT_FH_ percentages peaked in early adulthood and decreased slightly throughout aging (Figure 5B). Interestingly, aging correlated strongly with an increase in the percentage of effector cT_FH_ (CD4^+^CXCR5^+^CD27^+^CD45RA^−^) among total cT_FH_ (Figure 5C). Effector cT_FH_ are a subset of the total cT_FH_ population, and have shown to expand, as well as produce more of IL-21, in an antigen-specific manner following vaccination (31). There were no significant differences in cT_FH_ percentages between unstimulated and SEB stimulated conditions; however, younger and older pediatric participants had a significantly lower percentage of effector cT_FH_ than did adults under both unstimulated and SEB-stimulated conditions (Figure 5D). To address age-related differences between expression functional cT_FH_ markers, we explored IL-21, IL-2, CD154 (also known as CD40 ligand), ICOS (CD278), and TNF-α. T_FH_ expression of IL-21 and IL-2 play important roles in B cell maturation and isotype switching through transcriptional upregulation of BLIMP1 and AID (31). CD154 acts as a signal-two for B cell activation (32). ICOS interacts with ICOS ligand during cT_FH_ development (32). TNF-α-expression indicates a T_H_1-like cT_FH_ phenotype, which may lead to preferential development of antibodies more capable of inducing further cytotoxicity (33). Age-associated differences in effector cT_FH_ responses following SEB stimulation were less striking; however, younger pediatric participants showed significantly lower IL-21 and trending lower IL-2 expression than did adult participants (Figure 5E). Of note, higher net cT_FH_ expression of CD154 and TNF-α occurred in the older pediatric participants despite the lower percentage of effector cT_FH_ in this group compared to adults (Figures 5D,E). No significant differences have been observed in the levels of ICOS expression in effector cT_FH_ cells between children and adults. Similarly, gender had no significant impact on effector cT_FH_ populations or effector responses (Figures S4A–D in Supplementary Material).

### Unsupervised Comparison of Multifunctionality Between Age Groups

We used the cluster identification, characterization, and regression (CITRUS) tool to identify and further analyze differences in SEB stimulated CD8^+^ T cell responses between pediatric and adult participants (34). The analyses were performed by supervised gating of CD8^+^CD69^+^ T_EM_ and T_EMRA_ separated into adult (20–65 years old; *n* = 14) and young pediatric (6–15 years old; *n* = 11) participant groups. We then used CITRUS to perform unsupervised clustering based on MIP-1β, CD107a, TNF-α, IL-2, IL-17A, IFN-γ, and Granzyme B in abundance mode to distinguish cell signatures between pediatric and adult participants. We subsequently ran a predictive Nearest Shrunken Centroid (PAMR) association model with equal event sampling per file, using a minimum cluster size of 3% of the total events clustered and a cross-validation rate of 1.

**Figure 6:**
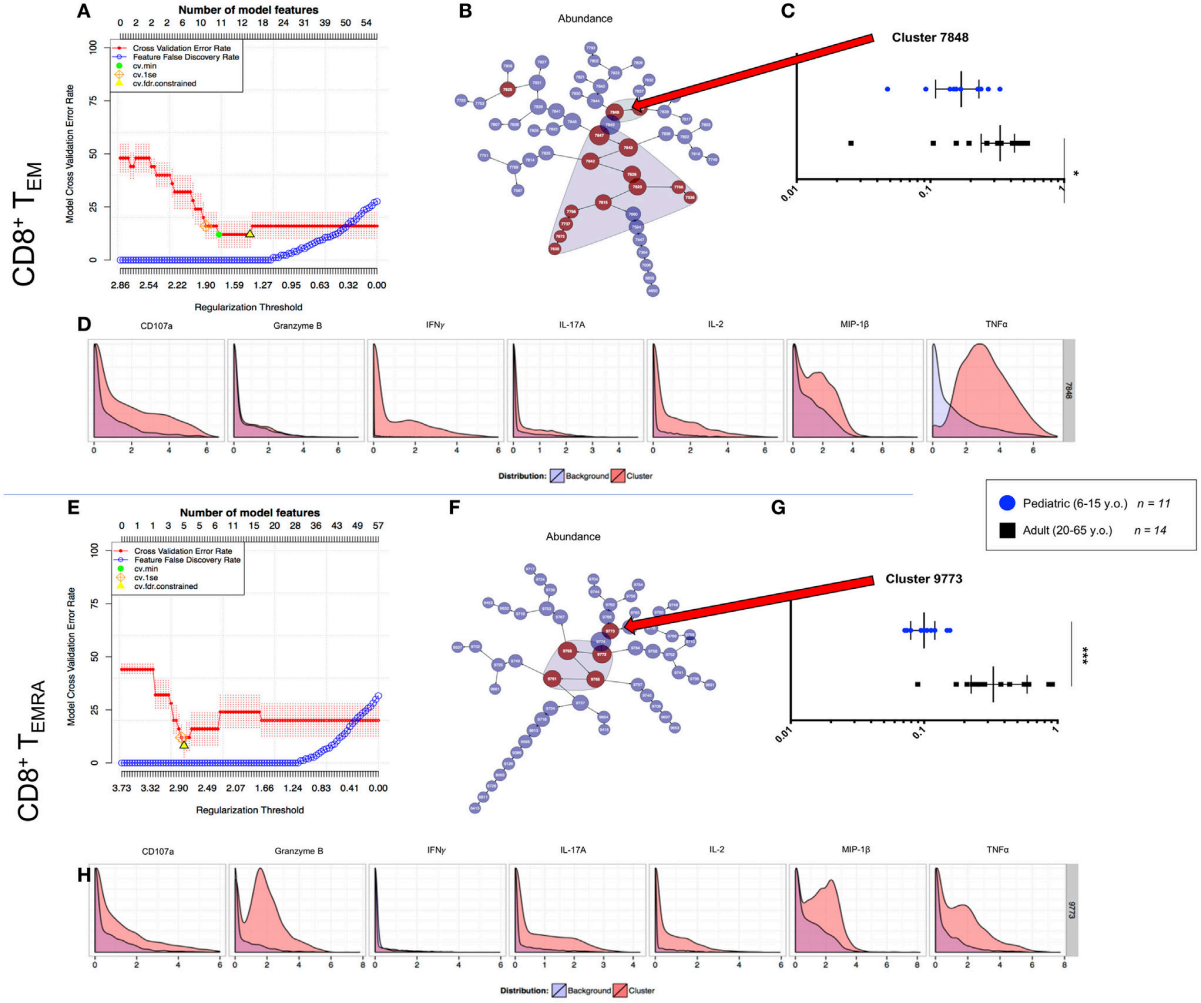
CITRUS analyses of peripheral multifunctional CD8^+^ T cell memory subsets. **(A,E)** Model error rate graphs showing false discovery rate constrained data points (yellow triangle—from which subsequent data are analyzed). This model analyzes the highest number of features (upper *x* axis) and the lowest cross validation error rate (red line) to determine the point at which the false discovery rate (blue line) increases. **(B,F)** Feature plots showing clusters of which significant differences (model features) between populations [i.e., 6–15-year-old pediatric (*n* = 11) and 20–64-year-old adult (*n* = 14) volunteers] are shaded. The clusters are nested from the center cluster, so the largest, most significant multifunctional clusters were chosen for analysis. The differences between the two populations within each of the chosen clusters for memory subsets **(C)** T_EM_, and **(G)** T_EMRA_ are shown, with each point in the box plot representing a separate participant. Values are a decimal equivalent of a percentage between 0 and 1, with median and interquartile ranges. Plot statistics were analyzed by Mann–Whitney test (*p < 0.05; ***p < 0.001). Chosen cluster features for each memory subset are shown **(D,H)** with the red histogram representing the effector expression within the chosen cluster and the blue histogram representing the average effector expression across the sampled population.

The resulting model error rate graphs for the activated CD8^+^ T_EM_ and T_EMRA_ analyses showed minimum cross-validation error rates below 20% and false-discovery rate constrained points with more than one model feature (Figures 6A,E). Nested clusters start with the largest, central most node, and branch into one or two child node(s), continuing until the maximum number of clusters greater than the minimum cluster size is generated. Feature plots showed clusters that were significantly different between analyzed groups (Figures 6B,F). To focus on multifunctionality, we analyzed the most multifunctional parent clusters that were significantly different between adult and pediatric groups: cluster 7848 for CD8^+^ T_EM_, and cluster 9773 for CD8^+^ T_EMRA_. The chosen clusters showed significantly lower abundance among pediatric participants than among adults (Figures 6C,G). We assessed multifunctionality by analysis of the CITRUS trees colored by channel, wherein heat maps indicate the relative intensity of each clustered effector on each node (Figures S5A,B in Supplementary Material). Further, histograms generated for the chosen clusters showed a highly multifunctional phenotype (in red) compared to background (originating parent node) expression in blue (Figures 6D,H).

Taken together, these analyses identified highly multifunctional activated CD8^+^ T_EM_ and T_EMRA_ populations that are significantly more abundant in adults than in children. We extracted these populations for analyses in order to validate our unsupervised data analyses and further explore the characteristics of these highly multifunctional cells.

### Downstream Identification of Cytotoxic and Proliferative Cellular Phenotypes From Within Highly Multifunctional CD8^+^ T Cell Populations

**Figure 7:**
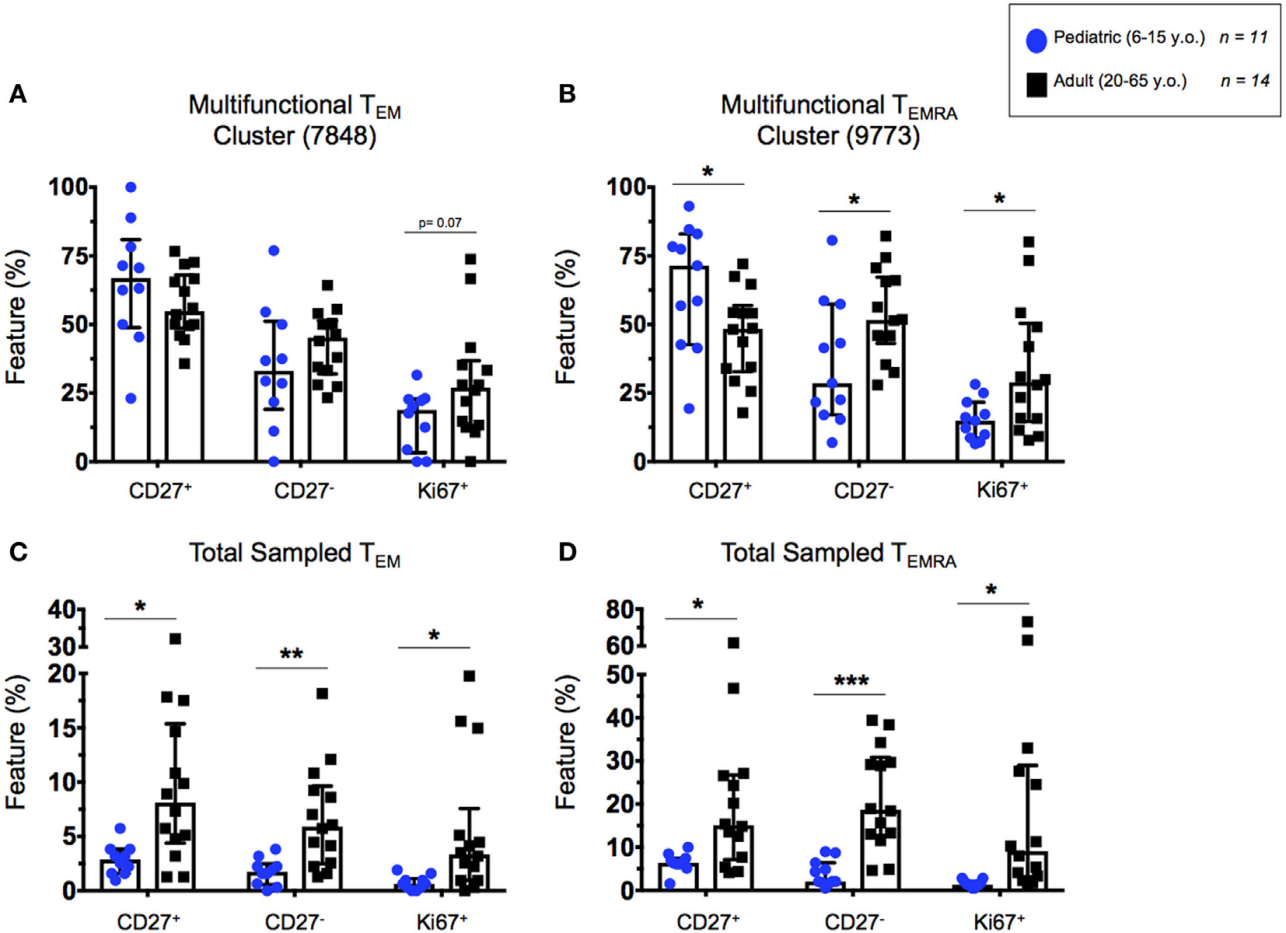
Highly multifunctional CD8^+^ T cell functional states. Scatter plots showing the percentages of highly multifunctional CD8^+^ T cells as defined by CITRUS node isolation. **(A)** T_EM_ and **(B)** T_EMRA_ cells within the chosen highly multifunctional nodes (see Figure 6) showing proportions of cytotoxic (CD27^−^) or proliferative (CD27^+^ and Ki67^+^) multifunctional CD8^+^ T cells in adults and children. **(C)** T_EM_ and **(D)** T_EMRA_ populations showing highly multifunctional CD27^+^. CD27^−^, and Ki67^+^ cells from within the total CITRUS sampled CD8^+^ memory T cell subsets, exhibiting the proportion of highly multifunctional cytotoxic or proliferative phenotypes from within the each memory subset. Populations split among 6–15-year-old pediatric (*n* = 11) and adult (*n* = 14) participants. Bars represent medians with whiskers indicating interquartile ranges. Scatter plot statistics were analyzed by unpaired *t*-test (*p < 0.05; **p < 0.01; and ***p < 0.001).

The previously described highly multifunctional CITRUS clusters 7848 (CD8^+^ T_EM_) and 9773 (CD8^+^ T_EMRA_), were exported as individual FCS files and analyzed by supervised gating using Winlist 9.0.1 software. We used CD27 expression to characterize CD8^+^ T cells as either generally cytotoxic and more mature (CD27^−^) or generally more naive (CD27^+^) phenotypes (28–30). Ki67 expression was used in this analysis to measure cell proliferation (35). We analyzed the correlation between CD27^+^ and Ki67^+^ (proliferating) T_EM_ and T_EMRA_ cells between pediatric and adult populations to determine whether T cell maturation trends varied between the groups. While Ki67^+^ levels are generally higher in adult T_EM_ and T_EMRA_, the trends between proliferation and CD27^+^ (more naive) cells were conserved between children and adults (Figures S6A–D in Supplementary Material). Cells from the highly multifunctional CD8^+^ T_EM_ cluster did not demonstrate significant differences in the percentage of CD27 expression between pediatric and adult participants (Figure 7A). However, there was a trend toward higher Ki67 expression in adult participants compared to children (*p* = 0.07; Figure 7A). In contrast, significant differences in CD27 and Ki67 expression levels were observed between children and adults in cells from the highly multifunctional CD8^+^ T_EMRA_ node (Figure 7B). Canonically, highly differentiated T_EMRA_ are thought of as being less proliferative (Ki67^+^) and more cytotoxic and multifunctional (CD27^−^) (30, 36). However, our data show that SEB stimulation can induce high levels of proliferation among cytotoxic CD27^−^ T_EMRA_ in adults, while T_EMRA_ in children retain a more naive-like state (CD27^+^).

Additionally, in order to normalize the observed phenotypes based on their presence within the total sampled memory populations (T_EM_ or T_EMRA_), we represented the total number of events from within the highly multifunctional clusters as a percentage of the total memory population sampled by CITRUS before clustering. These data confirmed our previous observations from CITRUS analyses that the pediatric participants exhibited a lower proportion of highly multifunctional CD8^+^ T_EM_ and T_EMRA_, compared to adults (Figures 6C,G). Further, we observed significantly higher total percentages of proliferative (Ki67^+^), cytotoxic (CD27^−^), and more naive (CD27^+^) phenotypes among the total activated CD8^+^ T_EM_ and T_EMRA_ populations in adult participants as compared to children (Figures 7C,D), indicative of the observation that events from within the multifunctional nodes are predominately made up of cells from adults.

## Discussion

Cell-mediated immune responses in healthy pediatric populations are a critical, yet underexplored, area of research, especially among school-aged children. SEB, a mediator of non-menstrual TSS, stimulates a robust, non-clonally specific, multifunctional T cell immune response in adult PBMC. In this study, we analyzed SEB-stimulated PBMC to better understand similarities and differences in the magnitude and functionality of pediatric and adult T cell responses.

T cell subset and memory population analyses are among the limited data sets available in pediatric samples. Previous publications have described an age-dependent increase among the proportion of CD4^+^ helper T cells (8, 9). Further, a greater proportion of naive T cells, and a lesser proportion of effector memory T cells, has been widely observed in children in both the lymphatic and circulatory systems (11). Our study reaffirms these findings among children and adults, even within a pediatric cohort that reaches into the mid-teenage years.

T cell activation, as defined by CD69 expression, occurs following engagement of the TCR and a costimulatory molecule. Superantigens such as SEB can engage both the TCR and CD28 homodimer as well as MHC class II molecules (1, 2), and are thus capable of inducing robust T cell activation. In this study, we show net CD69 expression of around 25% in both CD4^+^ and CD8^+^ adult peripheral T cells following SEB-stimulation, consistent with previous reports on non-clonal specific peripheral T cell activation (1–4). Interestingly, we show T cell activation in response to SEB to be age-dependent, with levels of net CD69 expression increasing to adult levels as children reach their mid- to late-teens.

T cell effector functions are diverse, including functions such as cytoxicity against infected targets, as well as recruitment, activation, and proliferation of many cell subsets (37). Superantigens can cause a dangerous and overwhelming proinflammatory T cell response that causes severe, sometimes fatal disease (3–6). Our data show that individual SEB-stimulated net CD8^+^ T effector memory responses are significantly higher in late-teens and adulthood than in younger pediatric participants. These age-related trends were not as strong regarding CD4^+^ effector memory responses. Interestingly, increases in net multifunctional T cell responses among both effector memory CD4^+^ and CD8^+^ cells were significantly correlated with age, with younger volunteers showing lower multifunctionality which increases through mid- to late-teenage years, before reaching adult levels. The significant increase of multifunctional T cells and CD8^+^ effectors in adults following SEB stimulation suggests a greater likelihood for the TSS-associated cytokine storm among adults compared to children, although the mechanism for these differences remains unclear.

Circulating T_FH_ are defined by CXCR5 expression. Although CXCR5 is expressed by activated CD4^+^ T cells, SEB stimulation has shown to be a poor inducer of CXCR5 expression (38), which we have confirmed in the present study. Interestingly, we show that aging through adulthood is significantly correlated with decreasing percentages of cT_FH_. In contrast, the percentages of effector cT_FH_ appear to increase throughout life. SEB-stimulation does not seem to play a significant role in the induction of activated cT_FH_, although it is able to stimulate effector responses within the activated cT_FH_ population. The only cT_FH_ effector function we observed to be strictly age related was IL-21 production, which has been shown to be important for induction of T_H_2- and T_H_17-associated immunoglobulin production following SEB stimulation (38). However, because the present study did not explore antibody responses to SEB stimulation among our participants, it is not possible to confirm these previously described findings.

Studies have shown that gender is an important contributing factor to immune variation in both children and adults (39), although the mechanisms behind these differences remain underexplored. Our data show higher CD4^+^ effector memory inflammatory cytokines in pediatric males, but few differences in CD8^+^ responses or CD4^+^/CD8^+^ T cell multifunctionality, following SEB stimulation. There were no significant differences in activation, defined by CD69 expression, between genders in any age strata. Further, no gender-associated differences were observed among cT_FH_ responses. Adult gender-associated human CMI studies have shown higher total T cell percentages in males and greater T cell activation (CD69^+^) in females following PHA-stimulation (40), as well as stronger cytotoxic transcriptional T cell responses in women after PHA stimulation followed by PMA-ionomycin restimulation (41, 42). Interestingly, our SEB-stimulated data did not recapitulate these observations among our equivalent-sized adult cohort. This heterogeneity among gender-associated T cell immune responses to various stimulation conditions is beyond the scope of this study, but merits further investigation, especially among more canonical antigen presentation models.

Dimensionality reduction tools are becoming more important in cytometric analysis, as the number of observable parameters within an individual experiment continues to increase. We have shown that CITRUS was able, without bias, to confirm and extend previously observed differences in the proportion of highly multifunctional responses between pediatric and adult participants. Further, we performed downstream analyses to evaluate cell activation and proliferative states in highly multifunctional subsets uncovered in our CITRUS analyses. These data confirmed previous results in adults showing that the proportion of CD27^−^ cells is higher in CD8^+^ T_EMRA_ than in T_EM_ (30). Additionally, our results showed that SEB stimulation can induce strong proliferation among these cytotoxic, more terminally differentiated adult CD8^+^ T_EMRA_, a population which is much less frequent in children. Dimensionality reduction allows for a more thorough probing of multivariate data, potentially uncovering trends that would otherwise be overlooked. CITRUS and/or other such dimensionality reduction tools need to be included in future studies directed to study multiple functions simultaneously.

Taken together, these findings support the notion that age, rather than gender, strongly influence the magnitude and functionality of SEB-stimulated T cell responses. Moreover, lower T cell activation in younger participants may intimate a possible mechanism for lower TSS-associated mortality in children compared to adults (7), and merits further investigation using clinical specimens from TSS patients. Finally, the data included in this manuscript suggest a critical need for in-depth comparisons of pediatric and adult T cell responses to MHC-restricted antigens. Understanding the variation within canonical immune responses in children and adults could play an important role in guiding the development of new, effective, vaccines designed for children.

## Ethics Statement

Peripheral blood mononuclear cell were collected from 20 healthy pediatric (6–17 years of age at the time of enrollment) and 14 healthy adult (20–65 years of age at the time of enrollment) volunteers, being recruited from the Baltimore-Washington area and the University of Maryland at Baltimore campus. These studies were approved by the University of Maryland at Baltimore Institutional Review Board (IRB) and were carried out in accordance with the Declaration of Helsinki. Written informed consent was obtained from all adult participants, as well as written assent and informed consent from the parents of any participant under the age of 18 years old—and assent from the pediatric participants themselves—prior to the conduct of any study procedures.

## Author Contributions

MR, MM, and MS designed the study, analyzed the data and wrote the manuscript; MR performed the experiments; LM aided in the statistical analyses; WC and RB contributed to the design, collected and processed the clinical samples, and helped draft the manuscript.

## Conflict of Interest Statement

The authors declare that the research was conducted in the absence of any commercial or financial relationships that could be construed as a potential conflict of interest.
