# Supplementary material for: Differences Between Pediatric and Adult T Cell Responses to In Vitro Staphylococcal Enterotoxin B Stimulation
Source: Front Immunol. 2018 Mar 20;9:498. doi: 10.3389/fimmu.2018.00498 (PMC5869216; doi:10.3389/fimmu.2018.00498)
Supplement: Supplementary file 1 [file presentation_1.pptx]

## Slide 1
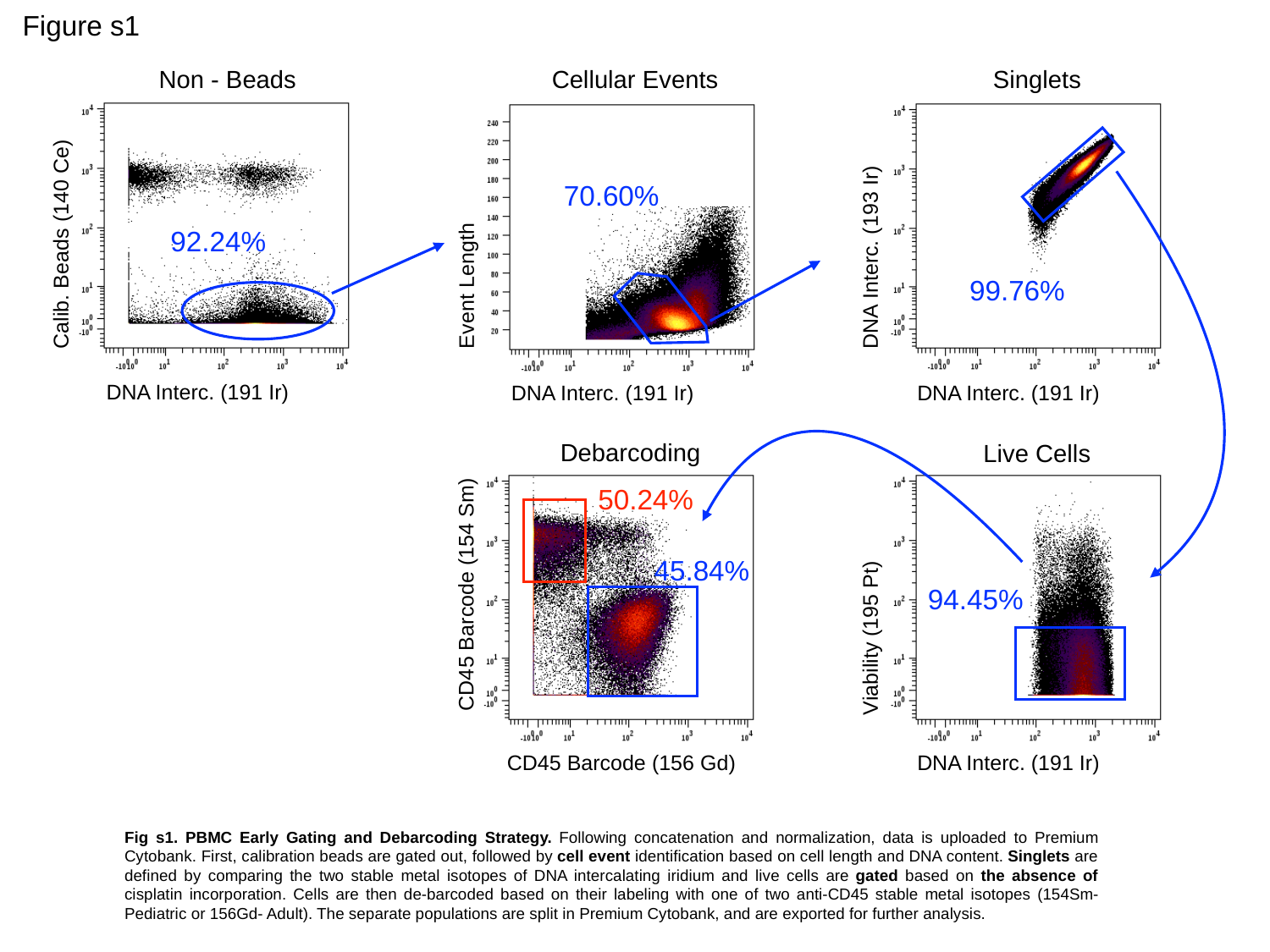

Figure s1
Cellular Events
Non - Beads
Singlets
99.76%
70.60%
92.24%
Calib. Beads (140 Ce)
Event Length
DNA Interc. (193 Ir)
DNA Interc. (191 Ir)
DNA Interc. (191 Ir)
DNA Interc. (191 Ir)
Debarcoding
Live Cells
50.24%
45.84%
94.45%
CD45 Barcode (154 Sm)
Viability (195 Pt)
CD45 Barcode (156 Gd)
DNA Interc. (191 Ir)
Fig s1. PBMC Early Gating and Debarcoding Strategy. Following concatenation and normalization, data is uploaded to Premium Cytobank. First, calibration beads are gated out, followed by cell event identification based on cell length and DNA content. Singlets are defined by comparing the two stable metal isotopes of DNA intercalating iridium and live cells are gated based on the absence of cisplatin incorporation. Cells are then de-barcoded based on their labeling with one of two anti-CD45 stable metal isotopes (154Sm- Pediatric or 156Gd- Adult). The separate populations are split in Premium Cytobank, and are exported for further analysis.
Representative experiment

## Slide 2
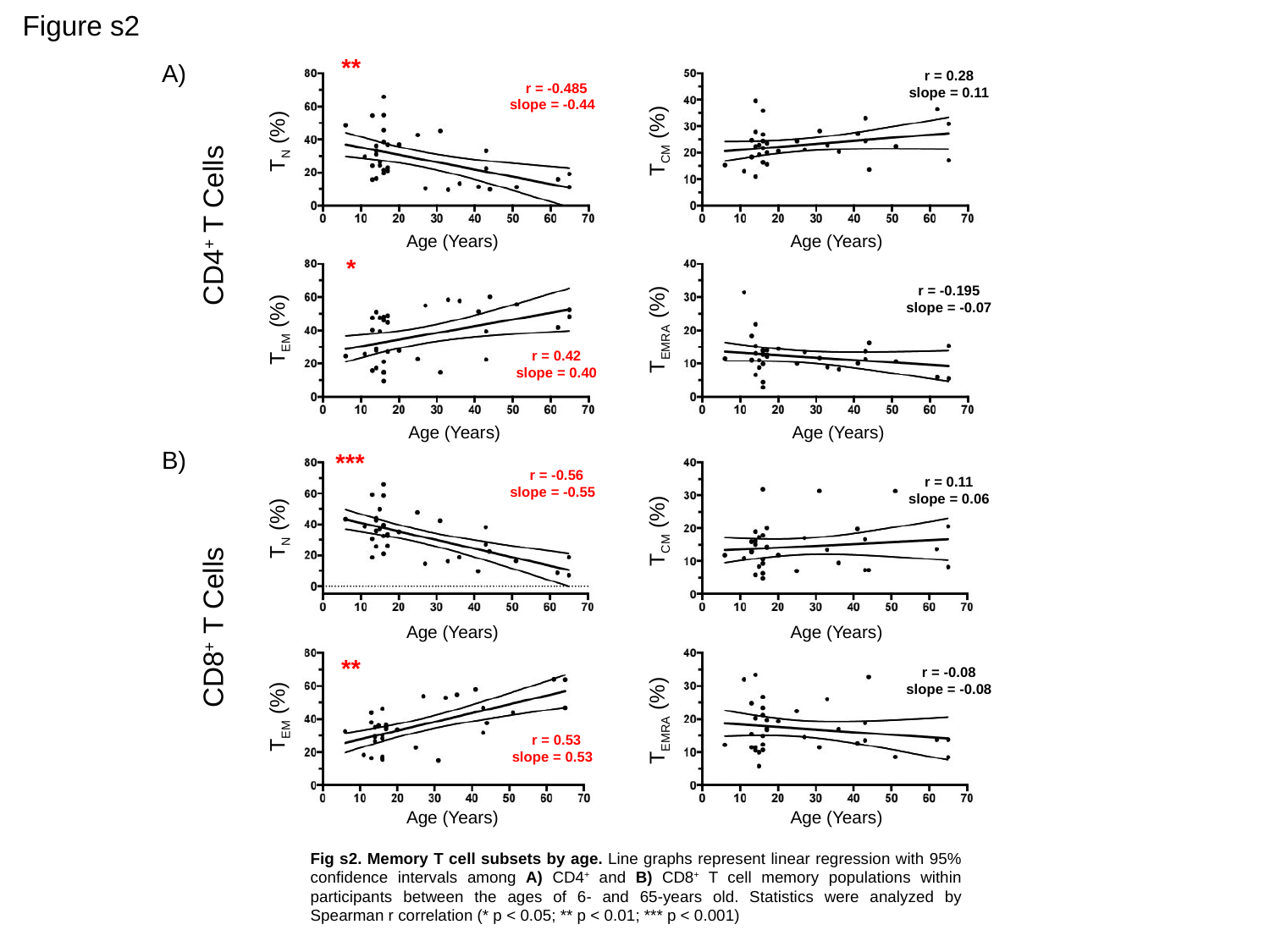

Figure s2
**
TCM (%)
TN (%)
TEMRA (%)
TEM (%)
r = 0.28
slope = 0.11
r = -0.485
slope = -0.44
CD4+ T Cells
*
r = -0.195
slope = -0.07
r = 0.42
slope = 0.40
***
r = -0.56
slope = -0.55
r = 0.11
slope = 0.06
CD8+ T Cells
**
r = -0.08
slope = -0.08
r = 0.53
slope = 0.53
A)
Age (Years)
Age (Years)
Age (Years)
Age (Years)
B)
TN (%)
TCM (%)
Age (Years)
Age (Years)
TEM (%)
TEMRA (%)
Age (Years)
Age (Years)
Fig s2. Memory T cell subsets by age. Line graphs represent linear regression with 95% confidence intervals among A) CD4+ and B) CD8+ T cell memory populations within participants between the ages of 6- and 65-years old. Statistics were analyzed by Spearman r correlation (* p < 0.05; ** p < 0.01; *** p < 0.001)

## Slide 3
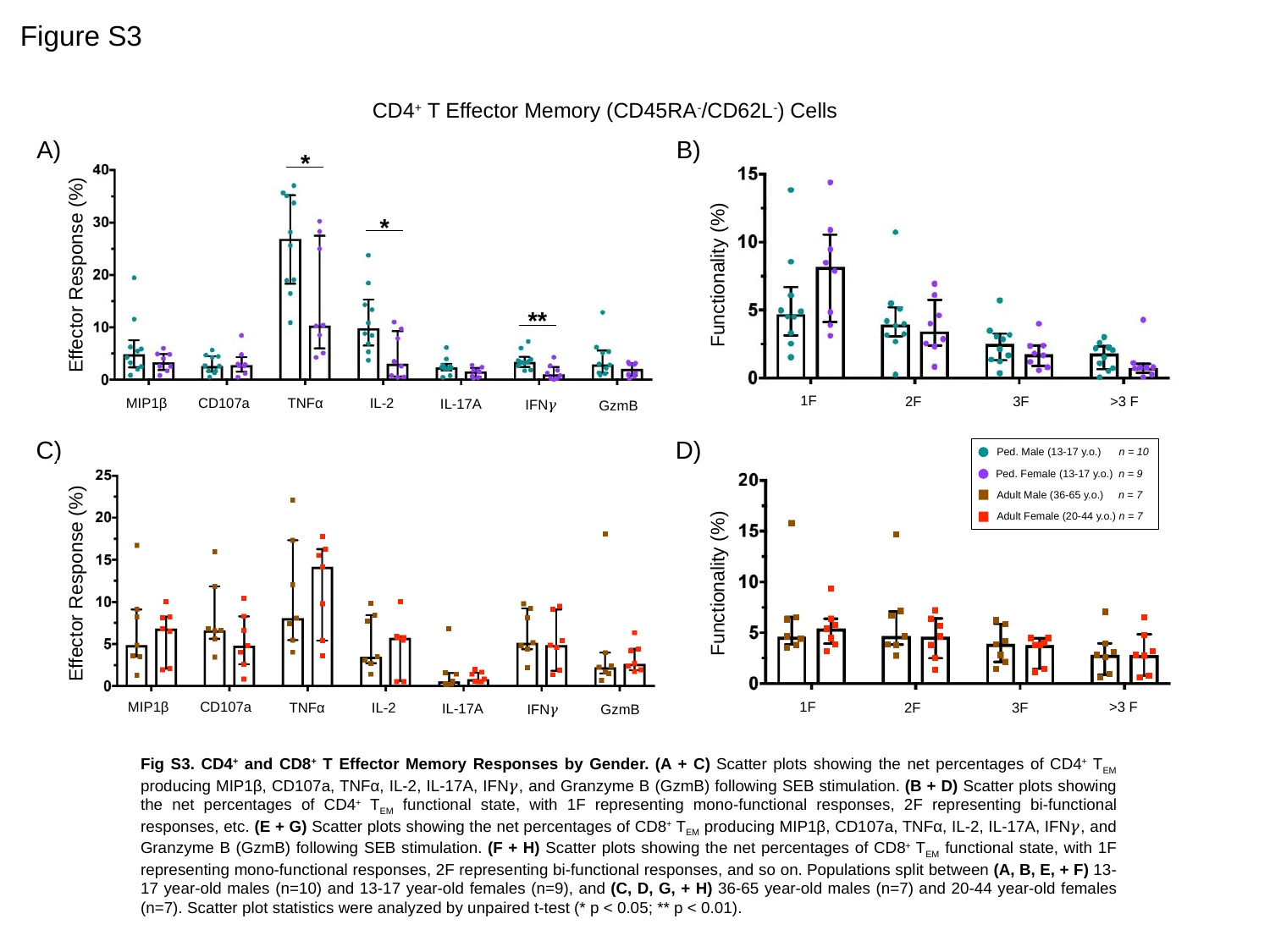

Figure S3
CD4+ T Effector Memory (CD45RA-/CD62L-) Cells
B)
A)
*
*
Effector Response (%)
Functionality (%)
**
1F
>3 F
2F
3F
MIP1β
CD107a
IL-2
TNFα
IL-17A
IFN𝛾
GzmB
D)
C)
Ped. Male (13-17 y.o.) n = 10
Ped. Female (13-17 y.o.) n = 9
Adult Male (36-65 y.o.) n = 7
Adult Female (20-44 y.o.) n = 7
Functionality (%)
Effector Response (%)
1F
>3 F
2F
3F
MIP1β
CD107a
IL-2
TNFα
IL-17A
IFN𝛾
GzmB
Fig S3. CD4+ and CD8+ T Effector Memory Responses by Gender. (A + C) Scatter plots showing the net percentages of CD4+ TEM producing MIP1β, CD107a, TNFα, IL-2, IL-17A, IFN𝛾, and Granzyme B (GzmB) following SEB stimulation. (B + D) Scatter plots showing the net percentages of CD4+ TEM functional state, with 1F representing mono-functional responses, 2F representing bi-functional responses, etc. (E + G) Scatter plots showing the net percentages of CD8+ TEM producing MIP1β, CD107a, TNFα, IL-2, IL-17A, IFN𝛾, and Granzyme B (GzmB) following SEB stimulation. (F + H) Scatter plots showing the net percentages of CD8+ TEM functional state, with 1F representing mono-functional responses, 2F representing bi-functional responses, and so on. Populations split between (A, B, E, + F) 13-17 year-old males (n=10) and 13-17 year-old females (n=9), and (C, D, G, + H) 36-65 year-old males (n=7) and 20-44 year-old females (n=7). Scatter plot statistics were analyzed by unpaired t-test (* p < 0.05; ** p < 0.01).

## Slide 4
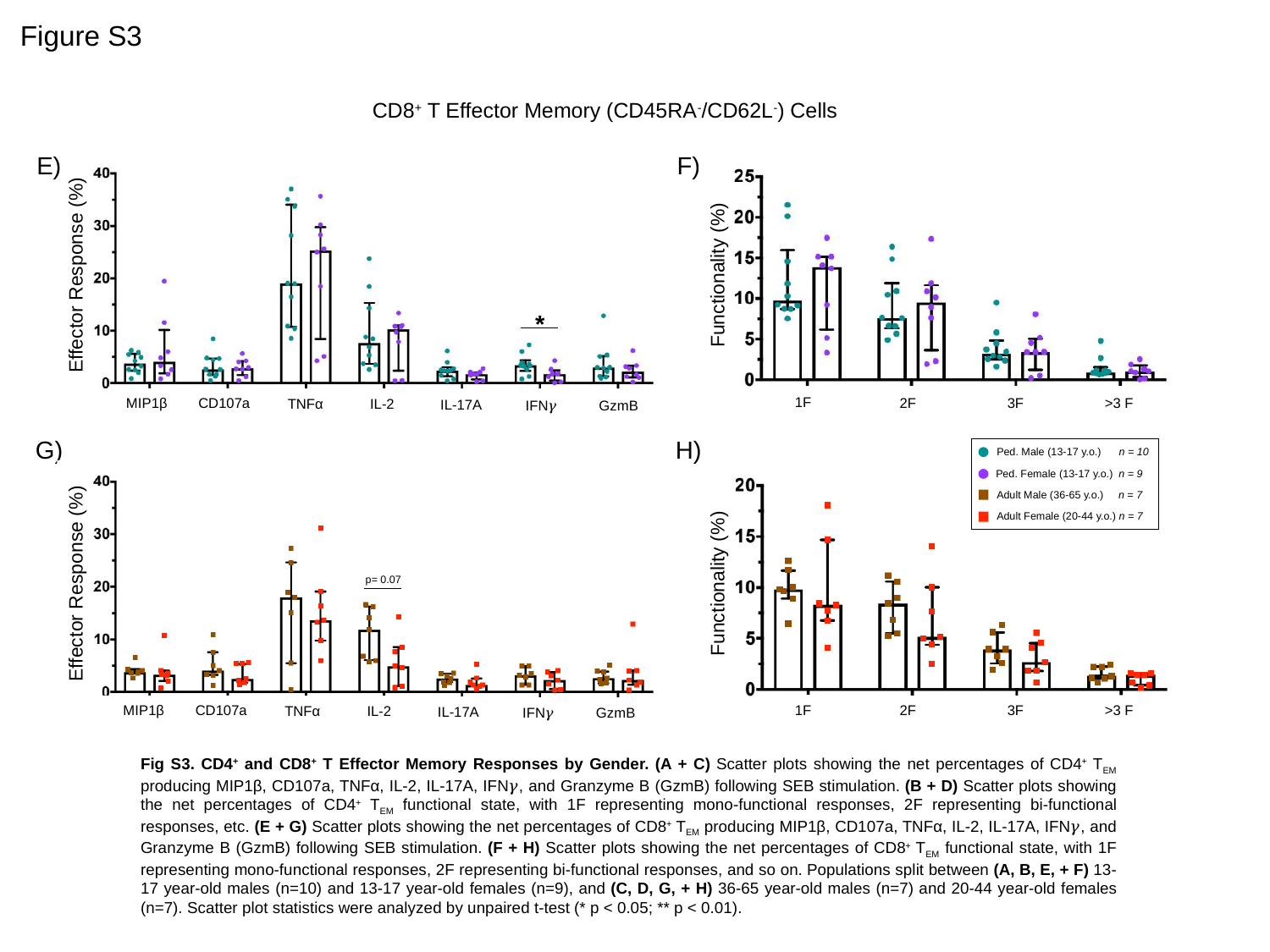

Figure S3
CD8+ T Effector Memory (CD45RA-/CD62L-) Cells
F)
E)
Effector Response (%)
Functionality (%)
*
1F
>3 F
2F
3F
MIP1β
CD107a
IL-2
TNFα
IL-17A
IFN𝛾
GzmB
H)
G)
Ped. Male (13-17 y.o.) n = 10
Ped. Female (13-17 y.o.) n = 9
Adult Male (36-65 y.o.) n = 7
Adult Female (20-44 y.o.) n = 7
Functionality (%)
Effector Response (%)
p= 0.07
1F
>3 F
2F
3F
MIP1β
CD107a
IL-2
TNFα
IL-17A
IFN𝛾
GzmB
Fig S3. CD4+ and CD8+ T Effector Memory Responses by Gender. (A + C) Scatter plots showing the net percentages of CD4+ TEM producing MIP1β, CD107a, TNFα, IL-2, IL-17A, IFN𝛾, and Granzyme B (GzmB) following SEB stimulation. (B + D) Scatter plots showing the net percentages of CD4+ TEM functional state, with 1F representing mono-functional responses, 2F representing bi-functional responses, etc. (E + G) Scatter plots showing the net percentages of CD8+ TEM producing MIP1β, CD107a, TNFα, IL-2, IL-17A, IFN𝛾, and Granzyme B (GzmB) following SEB stimulation. (F + H) Scatter plots showing the net percentages of CD8+ TEM functional state, with 1F representing mono-functional responses, 2F representing bi-functional responses, and so on. Populations split between (A, B, E, + F) 13-17 year-old males (n=10) and 13-17 year-old females (n=9), and (C, D, G, + H) 36-65 year-old males (n=7) and 20-44 year-old females (n=7). Scatter plot statistics were analyzed by unpaired t-test (* p < 0.05; ** p < 0.01).

## Slide 5
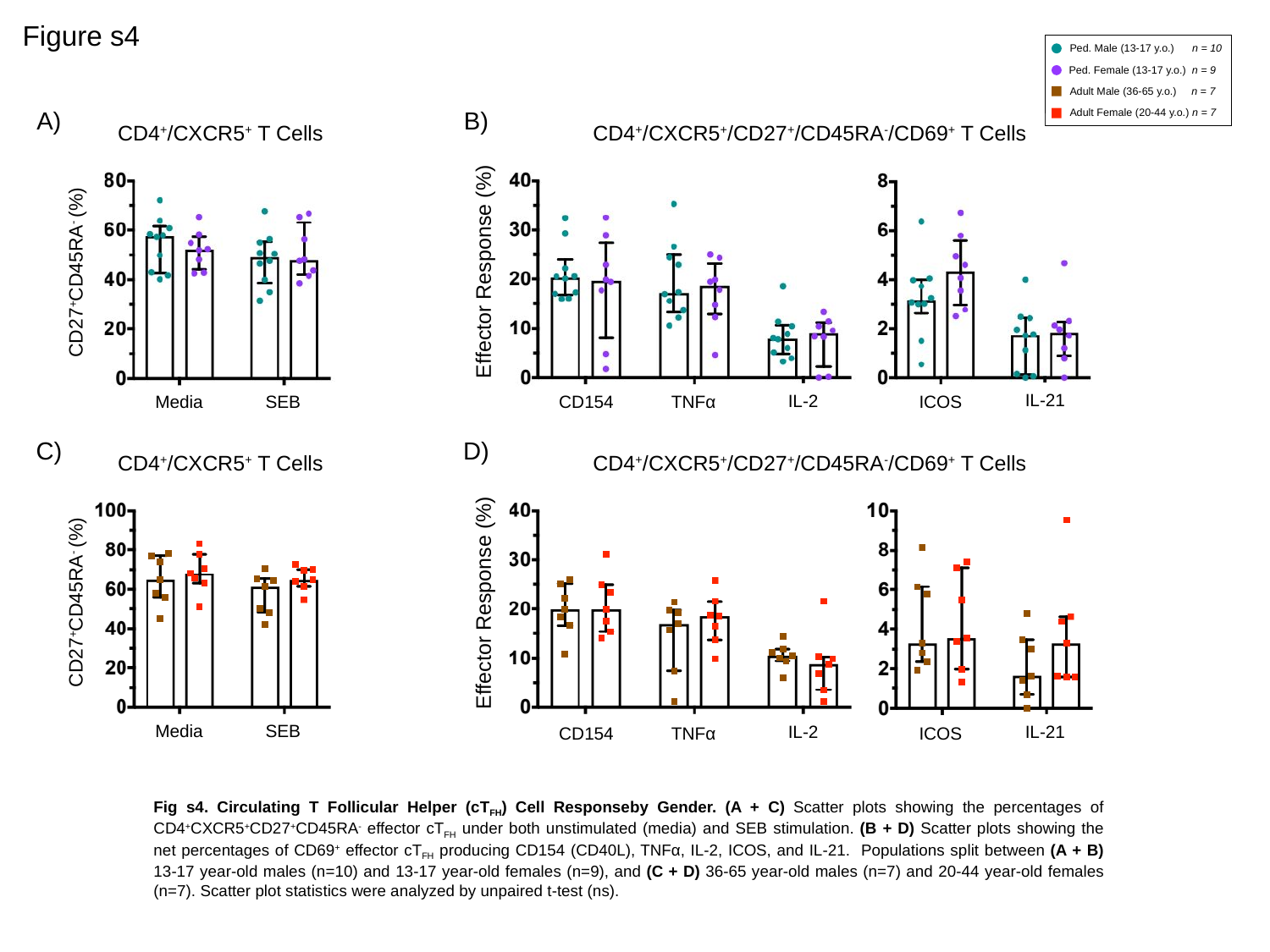

Figure s4
Ped. Male (13-17 y.o.) n = 10
Ped. Female (13-17 y.o.) n = 9
Adult Male (36-65 y.o.) n = 7
Adult Female (20-44 y.o.) n = 7
A)
B)
CD4+/CXCR5+ T Cells
CD4+/CXCR5+/CD27+/CD45RA-/CD69+ T Cells
Effector Response (%)
CD27+CD45RA- (%)
IL-21
IL-2
Media
SEB
TNFα
ICOS
CD154
D)
C)
CD4+/CXCR5+ T Cells
CD4+/CXCR5+/CD27+/CD45RA-/CD69+ T Cells
Effector Response (%)
CD27+CD45RA- (%)
Media
SEB
IL-21
IL-2
TNFα
ICOS
CD154
Fig s4. Circulating T Follicular Helper (cTFH) Cell Responseby Gender. (A + C) Scatter plots showing the percentages of CD4+CXCR5+CD27+CD45RA- effector cTFH under both unstimulated (media) and SEB stimulation. (B + D) Scatter plots showing the net percentages of CD69+ effector cTFH producing CD154 (CD40L), TNFα, IL-2, ICOS, and IL-21. Populations split between (A + B) 13-17 year-old males (n=10) and 13-17 year-old females (n=9), and (C + D) 36-65 year-old males (n=7) and 20-44 year-old females (n=7). Scatter plot statistics were analyzed by unpaired t-test (ns).

## Slide 6
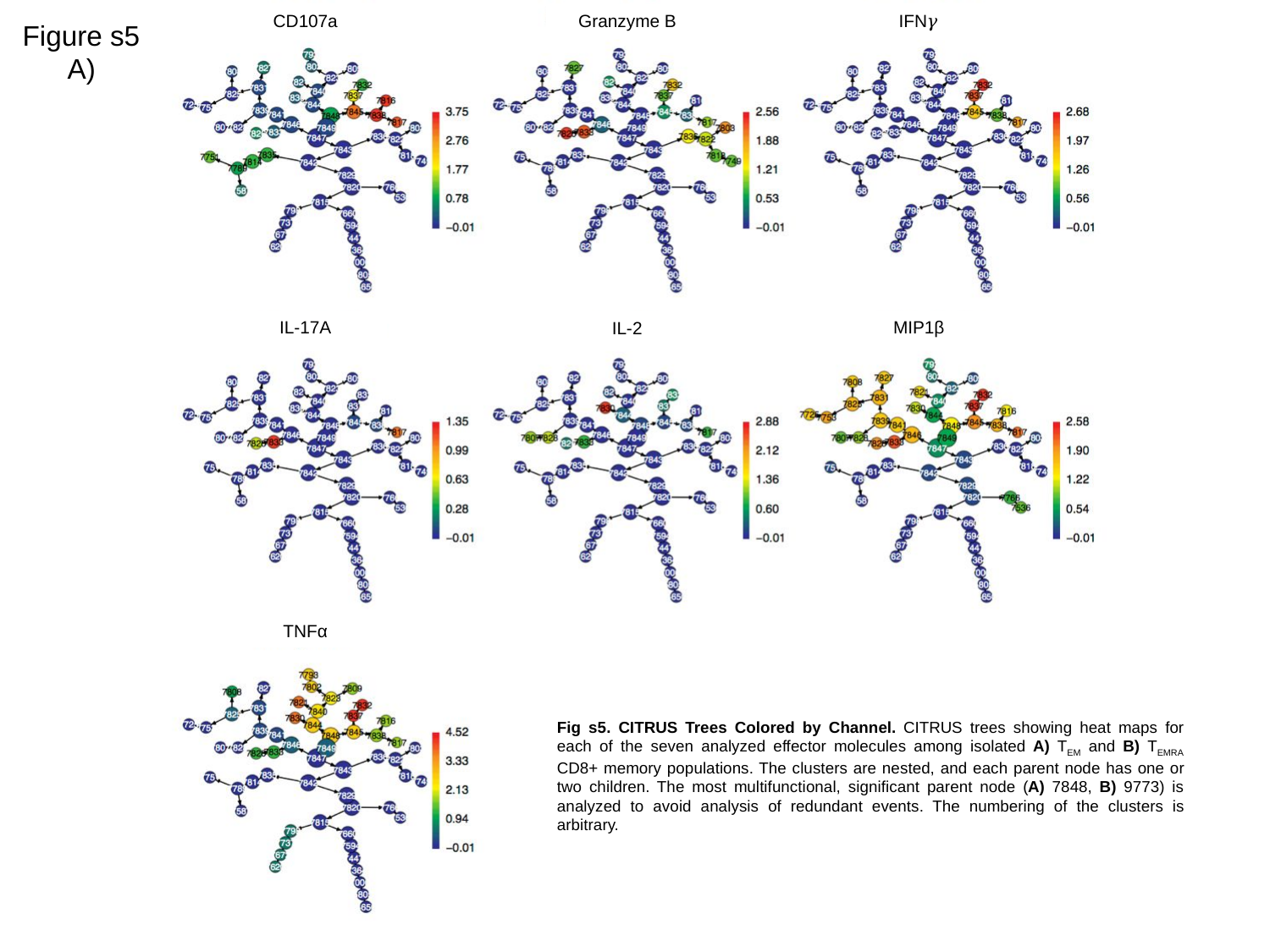

CD107a
IFN𝛾
Granzyme B
Figure s5 A)
IL-17A
MIP1β
IL-2
TNFα
Fig s5. CITRUS Trees Colored by Channel. CITRUS trees showing heat maps for each of the seven analyzed effector molecules among isolated A) TEM and B) TEMRA CD8+ memory populations. The clusters are nested, and each parent node has one or two children. The most multifunctional, significant parent node (A) 7848, B) 9773) is analyzed to avoid analysis of redundant events. The numbering of the clusters is arbitrary.

## Slide 7
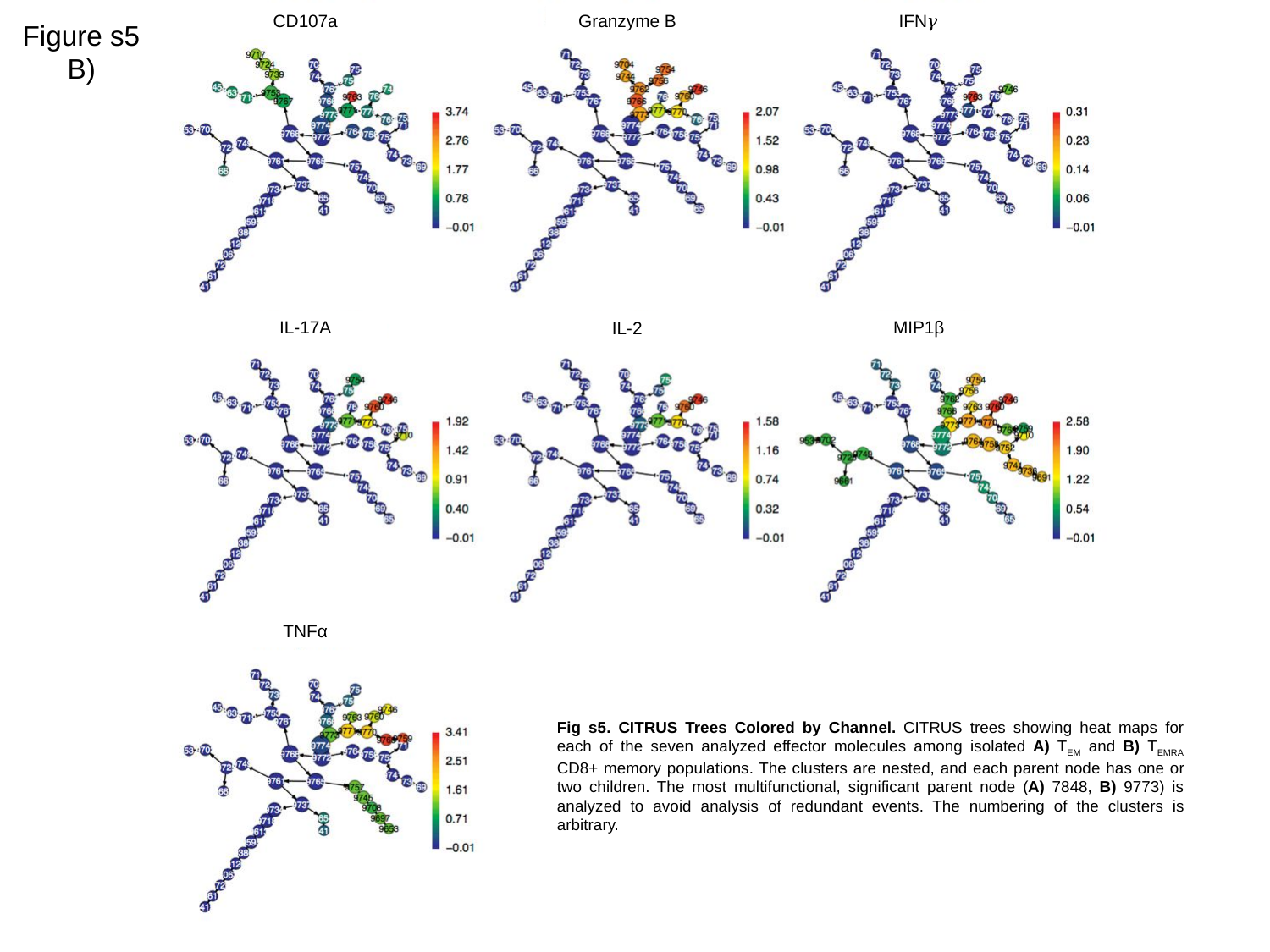

CD107a
IFN𝛾
Granzyme B
Figure s5 B)
IL-17A
MIP1β
IL-2
TNFα
Fig s5. CITRUS Trees Colored by Channel. CITRUS trees showing heat maps for each of the seven analyzed effector molecules among isolated A) TEM and B) TEMRA CD8+ memory populations. The clusters are nested, and each parent node has one or two children. The most multifunctional, significant parent node (A) 7848, B) 9773) is analyzed to avoid analysis of redundant events. The numbering of the clusters is arbitrary.

## Slide 8
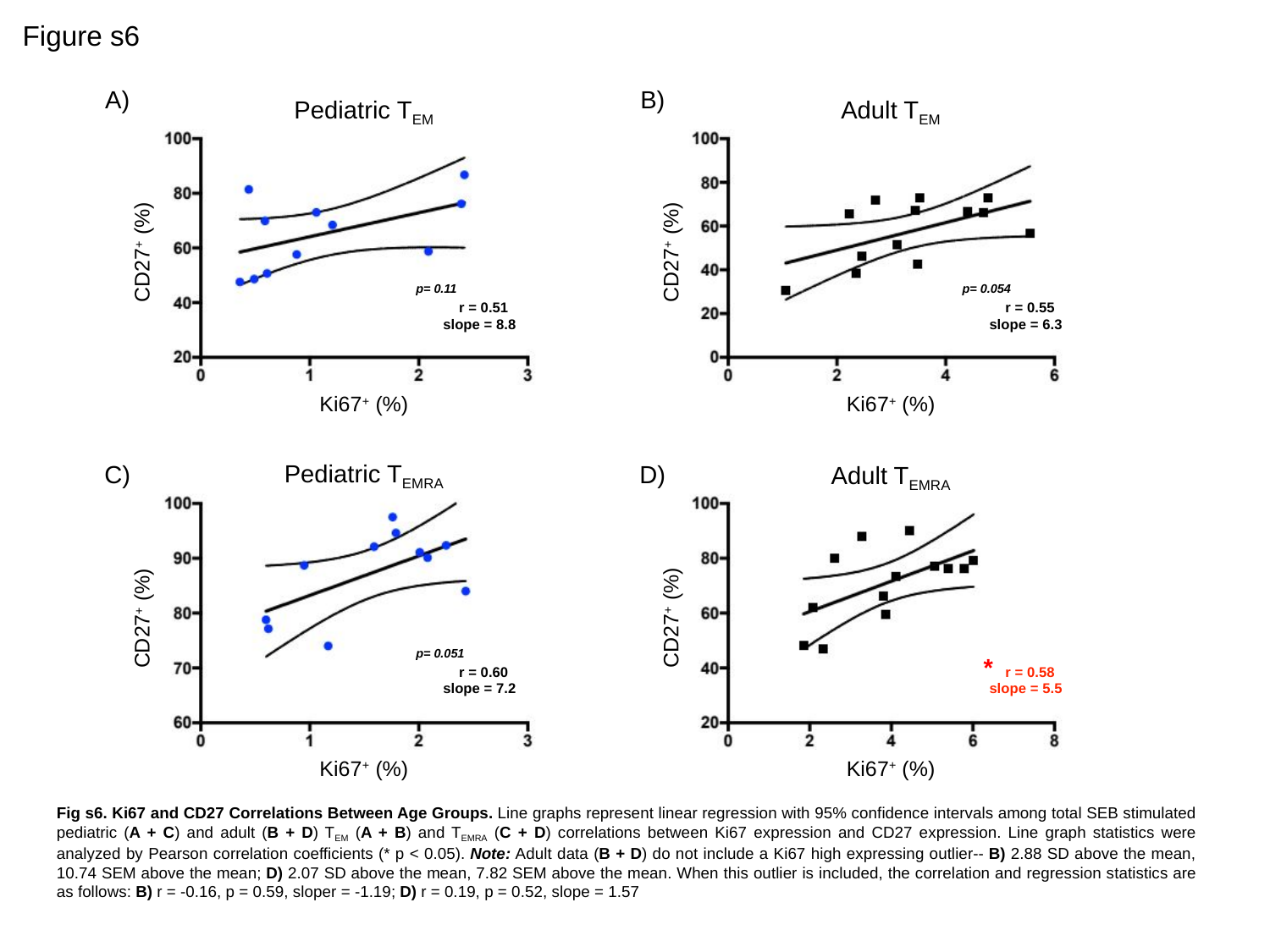

Figure s6
A)
B)
Pediatric TEM
Adult TEM
CD27+ (%)
CD27+ (%)
p= 0.054
p= 0.11
r = 0.55
slope = 6.3
r = 0.51
slope = 8.8
Ki67+ (%)
Ki67+ (%)
Pediatric TEMRA
C)
D)
Adult TEMRA
CD27+ (%)
CD27+ (%)
p= 0.051
*
r = 0.60
slope = 7.2
r = 0.58
slope = 5.5
Ki67+ (%)
Ki67+ (%)
Fig s6. Ki67 and CD27 Correlations Between Age Groups. Line graphs represent linear regression with 95% confidence intervals among total SEB stimulated pediatric (A + C) and adult (B + D) TEM (A + B) and TEMRA (C + D) correlations between Ki67 expression and CD27 expression. Line graph statistics were analyzed by Pearson correlation coefficients (* p < 0.05). Note: Adult data (B + D) do not include a Ki67 high expressing outlier-- B) 2.88 SD above the mean, 10.74 SEM above the mean; D) 2.07 SD above the mean, 7.82 SEM above the mean. When this outlier is included, the correlation and regression statistics are as follows: B) r = -0.16, p = 0.59, sloper = -1.19; D) r = 0.19, p = 0.52, slope = 1.57
